# Global transcriptional and miRNA insights into bases of heterosis in hybridization of Cyprinidae

**DOI:** 10.1038/srep13847

**Published:** 2015-09-08

**Authors:** Yi Zhou, Li Ren, Jun Xiao, Huan Zhong, Jun Wang, Jie Hu, Fan Yu, Min Tao, Chun Zhang, Yun Liu, Shaojun Liu

**Affiliations:** 1 Key Laboratory of Protein Chemistry and Developmental Biology of the State Education Ministry of China, College of Life Sciences, Hunan Normal University, Changsha 410081, China; 2 Guangxi Key Laboratory of Aquatic Genetic Breeding and Healthy Aquaculture, Guangxi Academy of Fishery Sciences, Nanning 530021, Guangxi, China

## Abstract

Hybrid *Megalobrama amblycephala* × *Culter alburnus* represents a population newly formed by interspecific crossing between two different genera. Here we assessed the expression pattern of mRNA and small RNA in newly formed F_1_, F_2_ and their progenitors. Large amounts of nonadditively expressed protein-coding genes showed parental expression level dominance (ELD). Interestingly, the ELD pattern could inherit from F_1_ to F_2,_ which guaranteed a stable appearance in progenies. The ELD-B genes were found to contribute to cell development, while the ELD-T genes were enriched in function of stress and adaptability. microRNAs (miRNA) also had similar expression patterns to genes. A high proportion of miRNAs showed nonadditive expression upon hybridization, and were found to target important genes with diverse roles potentially involved in stress adaption and development. Taken together, the gene and miRNA expression divergence contributes to heterosis in the newly formed hybrid, promising the successful existence of hybrid speciation.

Interspecies hybridization in teleosts promises advantage in development and adaptation, and is frequent in both artificial and natural environment. Several cultivated varieties, such as hybrid tilapia^1^, carp^2^, sturgeon^3^, Cichlid^4^ and salmonids^5^, are formed by interspecies hybridization as well. Meanwhile, interspecies hybridization also occurs in the natural environment^6^. However, interspecies hybridization is rare in nature due to the reproductive isolation among species. Upon successful hybridization, a progeny with improved adaptability and growth performance could be formed. These important phenotypic variations could result from the interaction between the two parental genomes, including allelic heterozygosity, and/or epigenetic changes, which result in changes at epigenetic, genetic, and gene expression levels^7^.

Hybrid *Megalobrama amblycephala* (♀, 2n = 48, BB) × *Culter alburnus* (♂, 2n = 48, TT) is a distant hybrid lineage (2n = 48, BT) from intercrossing between two different genera. Interestingly, both males and females of the F_1_ and F_2_ have normal gonadal development. Upon crossing the reproductive barrier, the hybrids show varieties and broader adaptability to stress challenges. Thus, it is an appropriate model for investigating heterosis in teleosts. On the other hand, interspecies hybridization is regarded as one of the most important evolutionary forces in speciation.

Previous study indicated that after hybridization, the progeny has often undergone unequal gene losses (genome fractionation), namely, one subgenome has more genes (dominant) than another (more fractionated)^8^. Usually, genes from the dominant subgenome are accompanied by higher expression levels. In teleosts, the genome dominance phenomenon has been reported for only few intraspecific cross hybrids, such as brook charr (*Salvelinus fontinalis*)^9^, rainbow trout (*Oncorhynchus mykiss*)^10^, and lake whitefish (*Coregonus* spp)^11^. Hybrids, with asymmetrical distribution of genetic loci, exhibit asymmetrical phenotypes of different subgenomes, which results in rapid evolution after hybridization. Meanwhile, hybridization is usually accompanied by gene loss. Furthermore, the rapid fusion of two different genomes triggers variation in gene structure, including alternative splicing, non-synonymous or even termination codon mutations^12^. Nevertheless, expression studies are needed to unveil the changes of the two subgenomes after the hybridization event. In intraspecific cross hybrids, demonstrating reciprocal differential loss of subgenome from two divergent fish lineages is difficult due to high homology. Therefore, interspecific hybrids provide a suitable solution. Artificial hybrids obtained in the laboratory from different genera via interspecific hybridization can facilitate epigenetic, genetic, and functional diversification studies of this process. In addition, the fertile progeny promises a consecutive model across generations. Hence, transcriptome studies of the nascent hybrid lines may reproduce the original genetic mechanism of natural hybrids and elucidate the molecular bases for hybrid vigor and adaptive traits.

Indeed, previous study has compared the gene expression patterns between hybrid fishes and their parents using Real-time quantitative PCR^13^. In another study, to determine nonadditive expression of genes in the hybrids, mid-parent value (MPV), the average expression value of the two parents, was employed^14^. The expression of genes which are significant different from MPV are demonstrated as nonadditive expression. Higher proportion of additivity compared to nonadditivity has been found in hybrids before^10^. However, the nonadditive responses in hybrids need explanation of the molecular mechanism for broader adaptability to stress challenges. Recent work suggested that partial genes are expressed as “expression-level dominance” (ELD) pattern, i.e. they are expressed equally compared to one progenitor and differently from the other^15^. However, the roles of these genes and the mechanisms behind this phenomenon remain unclear in hybrid fish. We aimed in the present work to determine the expression patterns and genetic as well as epigenetic bases for interspecies hybrid fish.

microRNAs (miRNAs), are endogenous ~22 nt small RNAs, which regulate gene expression at posttranscriptional levels via epigenetic modifications^16^. In interspecific hybrids, miRNA expression leads to nonadditive expression of target genes which may affect hybrid vigor and adaptive traits^17^,^18^. By cis- and trans-regulation, miRNAs control the gene expression underlying natural variation in metabolic pathways that influence broader adaptability to stress challenges. For instance, in *Squalius alburnoides*, a natural allopolyploid species, miRNAs promote genome stability and contribute to the evolutionary success of the hybrids^17^. Except for this pioneer report, studies assessing the miRNA profiles in hybrid fishes are scarce, while the heterosis may owe to miRNA expression changes during the hybridization process. The genome-wide gene expression bias of miRNA in hybrids is essential for heterosis. Also, the effects of miRNAs from different subgenomes on gene expression have not been illustrated yet. In hybrids, the miRNAs affecting genes may contribute to developmental changes and phenotypic variation in the newly formed organisms.

Existing findings have demonstrated that hybridization involves miRNA moderated gene regulation. However, the expression profiles of miRNAs derived from subgenome expression in hybrids are required. The present next-generation sequencing study would provide unprecedented resources to address such questions as how hybridization affects gene expression and changes the molecular pathways that could lead to broader adaptability in nascent hybrid fish, and whether miRNAs participate in this process. For this purpose, we performed RNA-seq and small RNA sequence in liver from F_1_ and F_2_ hybrid of *M. amblycephala* × *C. alburnus*, and their progenitors. Our data suggest a potential miRNA moderated regulatory mechanism for gene expression as bases for heterosis in hybridization.

## Results

### Formation of M. amblycephala × C. alburnus hybrids

**Figure 1:**
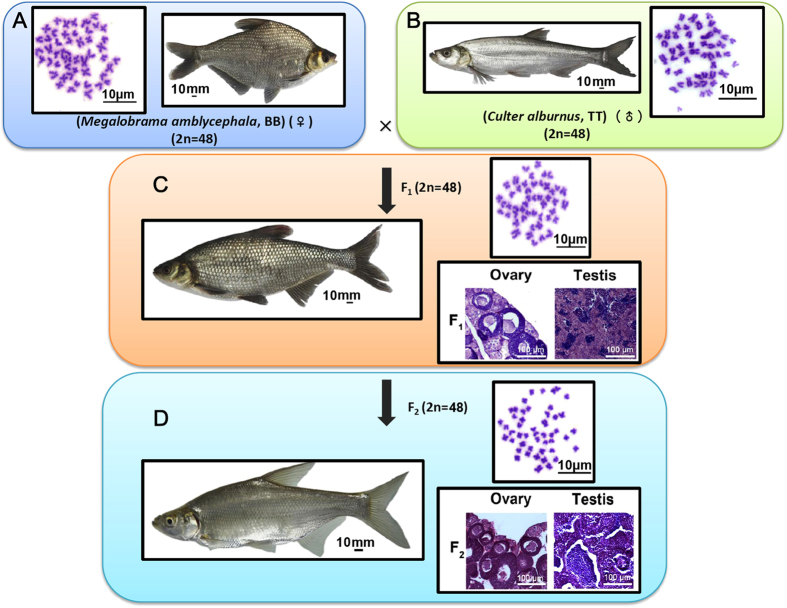
Formation of *M. amblycephala* × *C. alburnus* hybrids. (**A**) 48 Chromosomes were observed in *M. amblycephala*. (**B**) 48 Chromosomes were observed in *C. alburnus*. (**C**,**D**) After hybridization, F_1_ (**C**) and F_2_ (**D**) were obtained. The observation of chromosomes showed that no duplication of genome could found. The histology of gonads in hybrid showed fertile of these fishes. The photographs of the fishes were taken by Jun Xiao.

Hybrid fish were generated from female *M. amblycephala* × male *C. alburnus* (Fig. 1A,B). Two types of F_1_ hybrids were obtained in the present study, including diploid (2n = 48) and triploid (3n = 72) hybrids as previously reported^19^. In order to remove the polyploidy variable, we only used diploid hybrids (F_1_) for subsequent evaluation (Fig. 1C). Histology of gonads was performed and both mature oocytes and sperms were observed in F_1_ (Fig. 1C). Subsequently, diploid F_2_ individuals were obtained by self-mating of F_1_ (Fig. 1D). The diploid hybrids were bisexually fertile with normal development of gametes (Fig. 1D). Meanwhile, as previously reported^19^, for both diploid F_1_ and F_2_, 48 chromosomes (24 from *M. amblycephala* and 24 from *C. alburnus*) with no polyploidization, are suitable for further experimentation.

### RNA-sequencing in newly generated hybrids and progenitors

Four RNA-seq libraries from liver in adult F_1_ and F_2_, and their progenitors were constructed and sequenced. For convenience, hereafter, we will use both species names and their genome representations interchangeably, such as BB for *M. amblycephala* and TT for *C. alburnus*. After assembly, the unigenes were matched to public databases (Supplementary Table 1). To illustrate the gene function, we only used the genes which could be annotated by genome data of zebrafish for further analysis.

**Figure 2:**
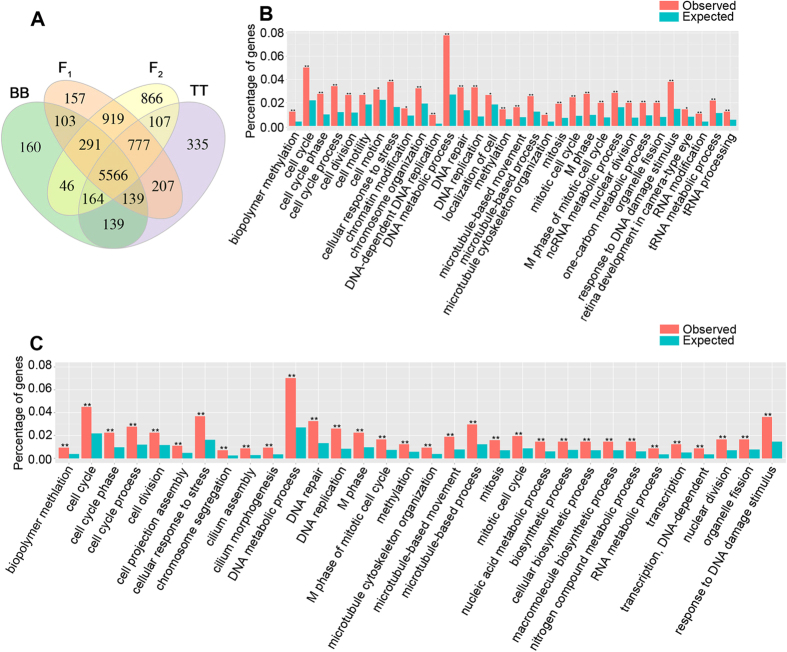
Global characterization of specific gene expression among *M. amblycephala*, *C. alburnus*, F_1_ and F_2_. BB, *M. amblycephala*, TT, *C. alburnus*. (**A**) Venn diagram analyses of specific genes in *M. amblycephala*, *C. alburnus*, F_1_ and F_2_. (**B**,**C**) Functional categories of specific genes in F_1_ (**B**) and F_2_ (**C**) except the 5566 co-expressed genes in all the samples. *P < 0.05 and **P < 0.01 to indicate the significant difference. Observed, indicated percentage of genes in the present study; Expected, indicated percentage of genes in the same category in GO enrichment analysis program.

Then, we BLAST the annotated genes of F_1_ and F_2_ against the unigenes of B and T reciprocally (Supplementary Fig. 1). We defined the genes except the 5566 co-expressed genes across all the samples as specific genes for each species (Fig. 2A). To compare expression quantities, the numbers of reads were normalized to relative abundance measured using fragments per transcript kilobase per million fragments mapped (FPKM)^20^. We also used Gene Ontology (GO) functional categories to identify gene function. The specific genes were identified by pairwise comparison among the species. In both F_1_ and F_2_, the specific genes (2593 in F_1_ and 3170 in F_2_) were significantly enriched for methylation, cell cycle, stress responses, and DNA and RNA modification (Fig. 2B,C). The specific genes in the two progenitors (1042 in BB and 1868 in TT) were significantly enriched for activities involving cellular macromolecules, oxidation-reduction, and activation of nucleic acids and proteins (Supplementary Fig. 2).

### Transcriptome differentiation among species during hybridization

**Figure 3:**
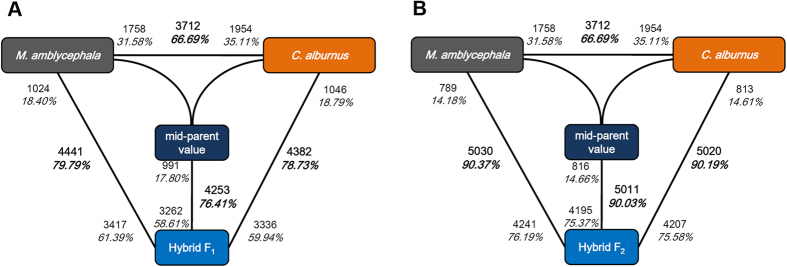
Nonadditively Expressed Genes in F_1_ and F_2_. BB, *M. amblycephala*, TT, *C. alburnus*. (**A**) Genes differentially expressed in F_1_ and their progenitors. (**B**) Genes differentially expressed in F_2_ and their progenitors. Numbers close to the species represent upregulated genes compared with the neighboring species. The number showed in bold indicates the total number of genes differentially expressed between two species.

Among all the expressed genes, 3712 (66.69%) were differentially expressed between *M. amblycephala* and *C. alburnus* (Fig. 3A,B). For the 3712 genes differentially expressed between the progenitors, 1758 (31.58%) showed higher expression levels in *M. amblycephala* (BB > TT), whereas 1954 (35.11%) were expressed at higher levels in *C. alburnus* (BB < TT). Compared with the progenitors, F_2_ showed higher number of differentially expressed genes than F_1_. For F_1_, 4441 (79.79%) F_1_-BB_dif_ genes were observed which were more than that of F_1_-TT_dif_ genes (4382, 78.73%) (P < 0.05, BH multiple test correction) (Fig. 3A and Supplementary Table 2). For F_2_, the numbers of F_2_-BB_dif_ (5030, 90.37%) and F_2_-TT_dif_ (5020, 90.19%) genes were almost equal (P > 0.05, BH multiple test correction) (Fig. 3B and Supplementary Table 2).

To identify nonadditively expressed genes, we compared the gene expression levels of F_1_ and F_2_ with MPVs calculated from the expression levels of the two parents. The result showed that the majority of hybrid F_1_ and F_2_ genes were nonadditively expressed genes. Only a minority of the differential expressed genes in F_1_ and F_2_ showed expression additivity (P < 0.05, BH multiple test correction). In the liver of F_1_, 4253 (76.41%) genes showed nonadditive expression (Fig. 3A and Supplementary Table 2). In F_2_, a higher proportion of genes (5011 genes, 90.03%) displayed nonadditive expression (Fig. 3B and Supplementary Table 2). In F_1_, the GO enrichment analysis of the nonadditive genes suggested enrichment for activators of nucleic acids, proteins and chromosomes as well as response pathways, including response to oxidation-reduction process and metal ion binding (Supplementary Dataset 1). These genes could therefore affect stress response. Interestingly, the nonadditive genes in F_2_ enriched for nucleic acid, protein and chromosome activities as well as oxidation-reduction process (Supplementary Dataset 1). In addition, nonadditively expressed genes were heritable across F_1_ and F_2_ (Supplementary Dataset 1). From F_1_ to F_2_, the number of nonadditively expressed genes increased (Fig. 3; Supplementary Dataset 1). The inherited nonadditively expressed genes enriched for metabolism, nucleic acid, and protein activities as well as oxidation-reduction process.

### Parental expression level dominance in hybrid progenies

**Figure 4:**
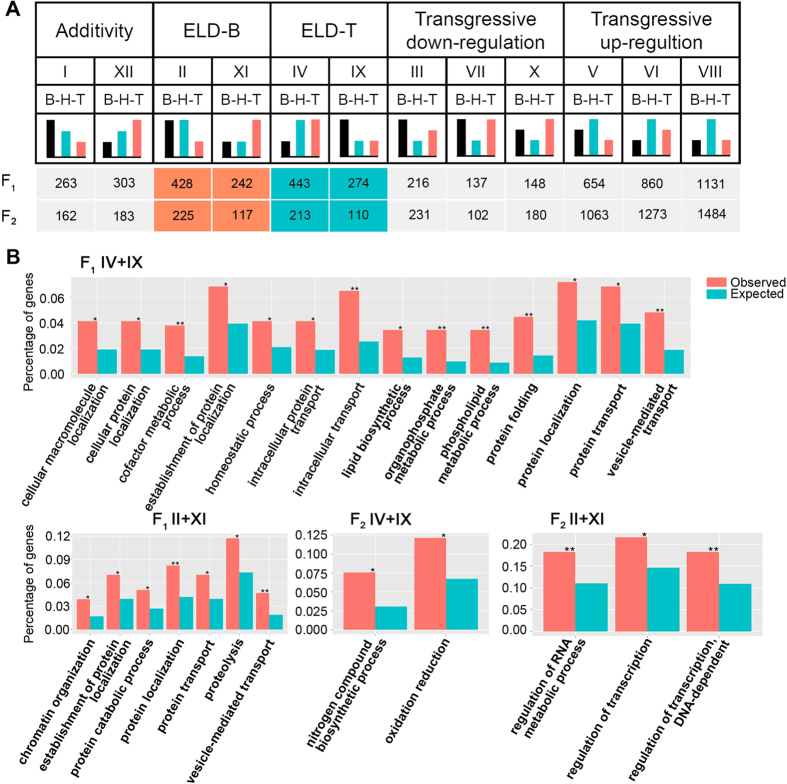
ELD of Genes in *M. amblycephala* × *C. alburnus* hybrids. (**A**) Twelve bins of differentially expressed genes. B, *M. amblycephala*, T, *C. alburnus*. H, hybrids. (**B**) Enriched GO terms of genes showing parental ELD in F_1_ and F_2_. *P < 0.05 and **P < 0.01 to indicate the significant difference.

In hybrids, ELD genes are those for which the expression level is statistically similar to one parent while different from the other. According to the previously described classification, we divided genes into 12 categories (as shown in Fig. 4A), based on differential expression patterns by comparing the hybrids to their parents as used in Rapp *et al.* (2009)^21^. We classified the ELD genes in F_1_ and F_2_ that showed similar expression with *M. amblycephala* as ELD-B genes; those with equivalent expression with *C. alburnus* were labeled ELD-T genes. In F_1_ and F_2_, 1387 and 665 genes showed parental ELD, respectively (Fig. 4A). In addition, both F_1_ and F_2_ showed no significant difference of gene number between ELD-B and ELD-T genes. Namely, these genes in the newly formed hybrids had no ELD bias toward the parents.

We then used GO analysis to pursue the possible functions of parental ELD genes in F_1_ and F_2_. In F_1_, ELD-B genes (II + XI) were enriched in genes for chromatin organization, protein activities and transport, while ELD-T (IV + IX) genes were mainly enriched for protein activities and lipid biosynthetic (P < 0.05, BH multiple test correction) (Fig. 4B). In F_2_, ELD-B (II + XI) genes were enriched for RNA activities, indicating distinct functions between the two groups of genes, whereas ELD-T (IV + IX) showed significant enrichment for nitrogen compound biosynthetic and oxidation reduction (P < 0.05, BH multiple test correction) (Fig. 4B).

**Figure 5:**
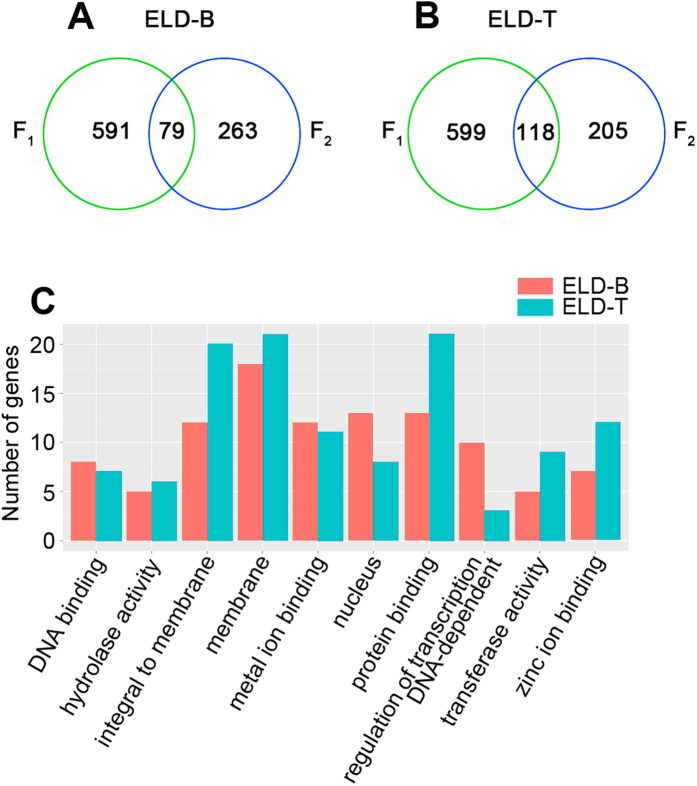
Heritable parental ELD genes in hybrids. (**A**) Venn diagram of ELD-B genes among F_1_ and F_2_. (**B**) Venn diagram of ELD-T genes among F_1_ and F_2_. (**C**) Enriched GO terms of heritable parental ELD genes in hybrids.

Meanwhile, the patterns of 79 ELD-B and 118 ELD-T genes were heritable across F_1_ to F_2_ (Fig. 5A,B). Subsequently, we assigned the heritable parental ELD genes into GO terms. Ten GO categories were found significant difference in these genes, including DNA binding, hydrolase activity, integral to membrane, membrane, metal ion binding, nucleus, protein binding, regulation of transcription DNA-dependent, transferase activity and zinc ion binding (Fig. 5C). The ELD-B set contained more genes for DNA binding, metal ion binding, nucleus and regulation of transcription DNA-dependent than the ELD-T genes. Interestingly, in the regulation of transcription DNA-dependent category, several genes involved in cell development were found, including cyclin D1, TNF receptor-associated factor 6 (TRAF6), signal transducer and activator of transcription 6 (STAT6), and period homolog 1a (per1a), suggesting the ELD-B genes may be mainly involved in the development of hybrids (Fig. 5C; Supplementary Dataset 2). By contrast, the other 6 categories, including hydrolase activity, integral to membrane, membrane, protein binding, transferase activity and zinc ion binding were more represented by ELD-T genes compared with their ELD-B counterparts (Fig. 5C; Supplementary Dataset 2). The zinc ion binding category, which is associated with disease and stress resistance, included zinc finger, DHHC domain containing 16a, tripartite motif containing 35–28 (trim 35–28), tumor necrosis factor, alpha-induced protein 3 (TNFAIP3), ring-box 1 (RBX1), E3 ubiquitin protein ligase, bloodthirsty-related gene family, member 32, DNL-type zinc finger, CREB binding protein and zinc finger, DHHC-type containing 18a. In addition, ELD-T genes also included some oxidation-reduction genes, such as WW domain containing oxidoreductase, Parkinson disease (autosomal recessive, juvenile) 2, parkin, oxoglutarate (alpha-ketoglutarate) dehydrogenase (lipoamide) and cholesterol 25-hydroxylase (Supplementary Dataset 2).

### Nonadditive expression of miRNAs and relevance to immunity and development

From the same tissues used for RNA-seq, 99 million small RNA sequencing reads were obtained. After removing adapter and miRNA confirmation, 155,204 miRNA sequences were matched to the Rfam database. We then filtered these candidate miRNAs with blast to the zebrafish miRNA database.

In total, 205 miRNAs in F_1_ were nonadditively expressed, while 215 were nonadditively expressed in F_2_ (Supplementary Dataset 3). In F_1_ and F_2_, 143 and 146 were nonadditively repressed miRNAs, respectively. Among these miRNAs, 111 nonadditively repressed miRNAs were inherited from F_1_ to F_2_, which indicated a conserved nonadditive repression in the hybrids (Supplementary Dataset 3). On the contrary, in F_1_ and F_2_, 62 and 69 were nonadditively activated miRNAs, respectively and 41 nonadditively activated miRNAs were inherited from F_1_ to F_2_ (Supplementary Dataset 3).

Several miRNAs with well characterized functions were found, e.g. immune responses and cell development (Supplementary Dataset 3). For nonadditively activated miRNAs, let-7d-5p, miR-155, miR-194a and miR-2188–5p are known to be involved in response to immune system. On the contrary, nonadditive repressive miRNAs, including let-7g, miR-125b-5p, miR-128-3p, miR-130b, miR-142a-3p, miR-152, miR-18c, miR-214, miR-27a-3p, miR-27c-3p, miR-29a, miR-301a, miR-30b, miR-363-5p and miR-93, participate in organism development. For example, miR-142a-3p^22^ and miR-27a-3p^23^ are key miRNAs in vascular development and blood mononuclear cell division. On the other hand, miR-214^24^, miR-29a^25^, miR-30b^26^, miR-363-5p^27^ and miR-93^28^ are involved in cell differentiation and development.

**Figure 6:**
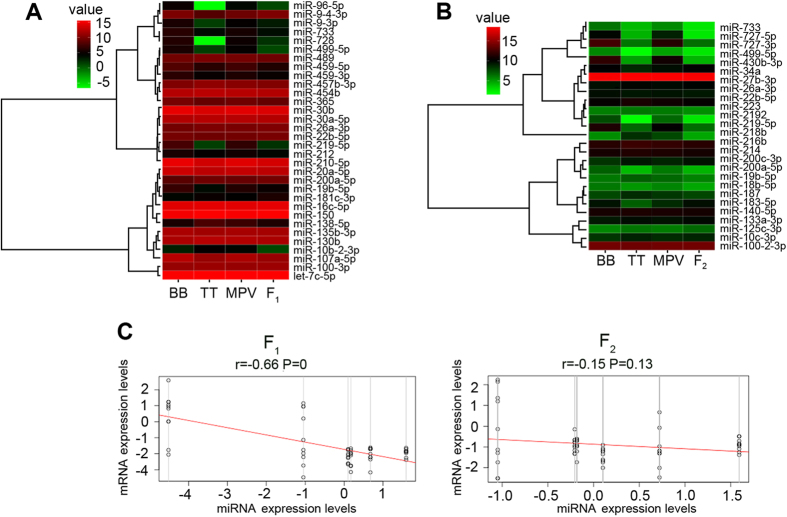
Effect of hybridization on expression of miRNAs. BB, *M. amblycephala*, TT, *C. alburnus*. (**A**) Nonadditive expression and miRNAs with parental ELD in F_1_ (only miRNAs which both attributed to nonadditive and parental ELD were showed in this Figure). (**B**) Nonadditive expression and miRNAs with parental ELD in F_2_ (only miRNAs which both attributed to nonadditive and parental ELD were showed in this Figure for concise). (**C**) Negative correlations between log2 fold changes of a subset of differentially expressed miRNAs and those of differentially expressed targets. Multiple genes are targeted by one miRNA which were indicated by vertical line. r, Pearson correlation efficient.

Similar with protein-coding genes, some miRNA also showed parental ELD in the newly formed hybrids. In F_1_, ELD-B (II + XI, 16), which was significantly less than those miRNAs showed ELD-T (IV + IX, 33) (Fig. 6A; Supplementary Table 3 and Supplementary Dataset 4). Similar results were obtained in F_2_ (ELD-B, 14 versus ELD-T, 20) (Fig. 6B; Supplementary Table 3 and Supplementary Dataset 4). Only 14 ELD miRNAs were inherited from F_1_ to F_2_, including several immune related and development regulatory miRNAs. Interestingly, miR-133a-3p^29^, miR-459-3p^30^ and miR-733^31^, which were reported for immune regulation, showed ELD.

The transcripts of target genes in F_1_ were negatively correlated with miRNAs (Pearson correlation, r = − 0.66, P = 0; Fig. 6C), while F_2_ showed little a negative correlation in F_1_ between transcripts of target genes and miRNAs (Pearson correlation, r = − 0.15, P = 0.13; Fig. 6C).

## Discussion

Hybrid fishes are widely distributed all over the world from artificial or natural interspecies hybridization. Upon crossing the interspecies barrier, the newly formed progenies display heterosis, such as fast growth appearance and broader adaptability to stress. Recent studies have focused on the molecular bases of hybridization, assessing gene expression and epigenetics. These findings suggested that heterosis may result from allelic interactions between subgenomes in hybrids with non-coding RNA, DNA methylation, and transcriptome changes^18^,^32^. In teleosts, several studies have shown the dynamic gene expression in rainbow trout^10^, Atlantic salmon^33^ and lake whitefish^11^,^34^. However, the epigenetic changes during hybridization which would aid to understand the possible molecular bases for heterosis in this widely distributed class are still unclear.

The ubiquitous hybrid fishes show advantages in evolutionary adaption, which confers them successful survival during the long time evolution. Meanwhile, interspecies hybridization and gene introgression with heterosis provide evolutionary force in teleosts. Most studies of hybrid teleosts have focused on cichlid fishes. It was reported that some new species of Cichlid are formed from the fusion between two old species^35^. The evolutionary force by hybridization results from the interactions between two different subgenomes in the newly formed hybrids. Thus, newly generated hybrids are good models to elucidate the heterosis and variation during hybridization. In addition, in teleosts, because there is no obvious mating barrier in some species, bisexual fertile hybrids could be obtained, which guarantees the tracing of generations for convenience. Therefore, multiple studies have investigated gene expression patterns in newly formed hybrid fishes^36^,^37^. In the present study, we used *M. amblycephala* and *C. alburnus* from different genera as progenitors. The newly formed hybrids were bisexual fertile. Thus, the high-throughput sequencing promises unprecedented opportunities to illustrate the dynamic genetic and epigenetic changes during this process.

Comparative genomics have indicated that during hybridization, the genome in progenies suffers a dynamic variation, including locus loss or duplication, which results in genome dominance, namely with genes from one subgenome having more loci and showing higher expression. These dominances in genotype and phenotype were well illustrated in intraspecific crossing hybrids^9^,^10^,^11^. In the present study, the interspecies crossed *M. amblycephala* × *C. alburnus* hybrid is suitable for studying genetic interaction among the two homoeologous genomes. In spite of its artificial formation, the improved vigor in the newly formed progenies may be due to genome dominance, also providing the genetic evidence and molecular bases for evolutionary forces accompanying hybridization. The newly formed F_1_ and F_2_ provided a trace line in the present study. The F_1_ presents the initial state after hybridization, while, F_2_ provides the subsequent melting of genomes in the following generation, indicating continuous interaction between the subgenomes. Large amounts of nonadditively expressed genes were found in both F_1_ and F_2_, which suggested unstable genomes in progenies. Meanwhile, some nonadditively expressed genes expressed across generationally also showed conservation of the genetic changes in hybrid descendants, indicating that they may contribute to heterosis in these organisms. Meanwhile, the nonadditively expressed genes were readily identified in different hybrid fishes, proving the widespread presence during hybridization.

Among the nonadditively expressed genes, parental ELD genes were found in newly formed hybrid fishes. Numerous studies have reported that these parental dominant models affecting the genotypes at the mRNA level underlie heterosis in hybrids. In the present study, 1387 and 665 ELD genes were found in F_1_ and F_2_ hybrids, respectively, representing a minority of the genes in hybrids. Despite their minority, the identified parental ELD genes in F_1_ and F_2_ suggest indispensable functions in these organisms. The ELD-B set contained more genes for DNA binding, metal ion binding, nucleus and regulation of transcription DNA-dependent than ELD-T; while hydrolase activity, integral to membrane, membrane, protein binding, transferase activity and zinc ion binding were more represented in ELD-T than ELD-B genes. In addition, we found that the ELD-B genes participate in cell development, while ELD-T set is involved in oxidation-reduction as well as disease and stress resistance.

In *Squalius alburnoides*, a hybrid ploidy teleost, the variation of miRNA expression levels with nonadditive expression was shown to be related to cellular functional stability^17^. Despite the ploidy, the heterogeneous genetic materials in hybrids influence the expression of miRNA and genes, affecting the phenotypes. Similarly, we found that several miRNAs showed nonadditive expression in F_1_ and F_2_ individuals. The substantial number of miRNAs may play a crucial role in heterosis for newly formed lines like *Squalius alburnoides*. Here, several nonadditively expressed miRNAs, similar to mRNA, showed parental ELD expression in F_1_ and F_2_, which indicated similar molecular mechanisms in the hybrids. For nonadditively expressed miRNAs, nonadditively activated miRNAs were mainly involved in response to immune system, while nonadditively repressed miRNAs were mostly involved in organism development. Thus, these functional miRNAs with nonadditive expression indicate that miRNAs are plausible regulators during hybridization, and cause differential expression of protein-coding genes in hybrids. Also, the variation of their expression suggests that miRNAs are possible key regulators that are critical for immune response and adaptation in hybrids. The negative correlation between miRNAs and target genes were significant in F_1_ rather than F_2_. This could due to the genetic changes in F_2_. The details of this variation need to be elucidated in further studies.

**Figure 7:**
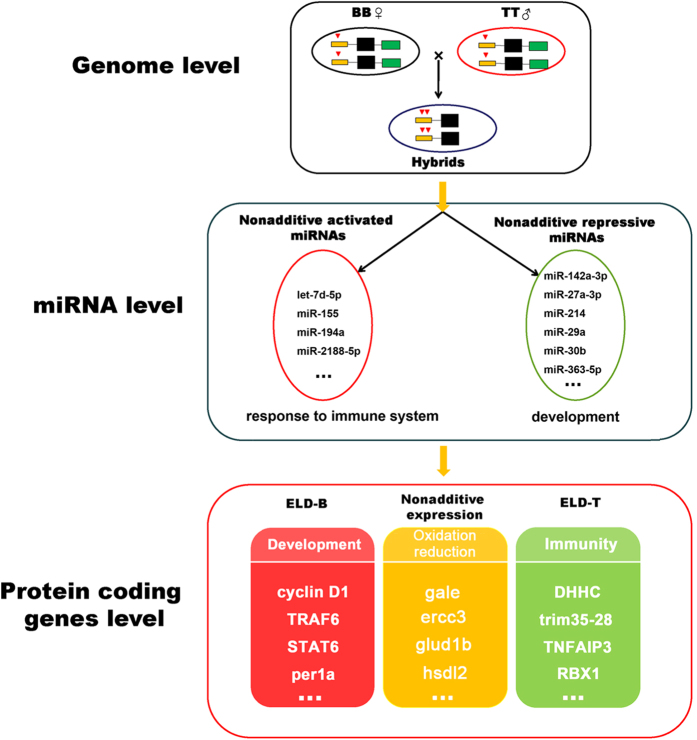
A miRNA-transcripts model for Expression Regulation during hybridization. (**A**) Genome level. Duplication (indicated by red triangles) and deletion (indicated by green bars) of loci in genome during hybridization. (**B**) miRNA regulation level. Nonadditively expressed miRNAs target genes involved in response to immune system and development. (**C**) Expression levels of protein-coding genes. ELD genes and nonadditively expressed genes contribute to the heterosis in newly formed hybrids.

The new hybrid *M. amblycephala *×* C. alburnus* lines, from different genera with the B and T genomes, indicated a genetic melting of organisms, exhibiting improved adaption to stress. Meanwhile, this heterosis was inheritable from F_1_ to F_2_, which guarantees a stable appearance in progenies. These ameliorative and hereditable phenotypes result from genome variation, and cause miRNA and mRNA expression changes; these findings propose a gene regulatory scheme for hybrids. We showed a dynamic regulation of homoeologs in newly formed hybrids, and demonstrated the miRNA regulated gene expression in the melting genomes (Fig. 7). We propose three different levels for heterosis that includes improved stress and adaptability during hybridization. First, the interaction between subgenomes from different species leads to gene loss or duplication as well as DNA modification such as methylation in the newly formed genome. Second, nonadditively expressed miRNAs affect target genes that in turn yield the improved stress and development. Third, for protein-coding genes, the expression of ELD-B genes may contribute to cell development, while that of ELD-T genes are involved in stress and improved adaptability. Taken together, the expression changes of miRNA and mRNA in hybrids showed stable variation during the early hybridization, and were passed down from generation to generation, which accounts for the successful advent of interspecies fishes, providing evolutionary force in natural population.

## Methods

### Fish cross and sampling

All experiments were approved by Animal Care Committee of Hunan Normal University and followed guidelines statement of the Administration of Affairs Concerning Animal Experimentation of China. *M. amblycephala* and *C. alburnus* strains utilized in the present study were obtained from the Engineering Research Center of Polyploid Fish Breeding and Reproduction of the State Education Ministry, located at Hunan Normal University, Changsha, China. The protocols for crosses and culturing were described previously^19^. All selected individuals were adults (24 months-old). Liver tissues were excised from animals of each experimental group and stored in RNAlater (Ambion Life technologies, Grand Island, NY, USA) at −80 °C.

### RNA isolation, cDNA library construction and sequencing

Total RNA was purified from homogenized tissue samples using RNA Trizol (Invitrogen, Carlsbad, CA, USA) and quantified with Agilent 2100 Bioanalyzer (Agilent, Santa Clara, CA, USA). mRNAs were isolated by employing magnetic beads with Oligo (dT). The DNase I reaction was performed at 37 °C for 15 min and ended with addition of 2.5 mM EDTA by incubation at 65 °C for 10 min. The mRNA, mixed with fragmentation buffer, was fragmented and used for cDNA library construction. Four cDNA libraries representing each kind of fish were constructed. Each sequencing library was prepared from a mixed pool of 500 ng of mRNA from four individuals, for a total of 2 μg. The cDNA libraries were synthesized using the mixed mRNA fragments as templates with the TruSeq RNA sample preparation kit V2 (Illumina, San Diego, CA, USA) according to the manufacturer’s instructions. The libraries were sequenced using Illumina HiSeq 2000, at BGI Shenzhen, Guangdong, China.

### *De novo* assembly and annotation

The generated raw reads were first filtered by removing the adaptors. The *de novo* assembly was carried out with Trinity^38^ using default parameters, to generate contigs and unigenes. In the present study, unigenes were further evaluated. Blastx alignment (*E value* < 0.00001) between unigenes and protein databases like NR, Swiss-Prot, KEGG and COG was employed to annotate the genes. The best aligning results were assigned as the annotation of the unigenes. In case of conflicting results among the databases, the priority order was defined as NR, Swiss-Prot, KEGG and COG. If the unigenes could not be aligned to all databases, ESTScan v3.0.2^39^ was used to decide sequence annotation. The Read-mapping was processed by Burrows-Wheeler Aligner using the transcriptome of progenies as references^40^.

### Differential expression analysis

The FPKM method^41^ was used to calculate the relative expression levels. The adjusted *P-values* were used in false discovery rate (FDR) for multiple hypothesis testing, which deploys the Benjamini-Hochberg correction to determine difference significance between samples. Using the reads from each group (*M. amblycephala*, *C. alburnus*, F_1_ and F_2_), the relative abundance of transcripts was calculated for each unigene in the clusters of the assembled unigenes. The unigenes with FDR ≤ 0.001 and fold change >2 were considered as differential expression genes, and submitted to further analysis.

Gene Ontology was used to illustrate the functional annotation of the differential expression genes among samples. GO enrichment analysis was carried out with the Blast2GO v2.5.0 software^42^. The GO terms with FDR < 0.05 were considered as significantly enriched.

### Small RNA library construction and sequencing

Four small RNA libraries were obtained from the same tissues used for cDNA libraries. After total RNA quality control testing, each small RNA library was prepared from a pool of 250 ng total RNA from each of the four individuals, a total of 1 μg. Then, small RNA sequencing was carried out as previously on Illumina HiSeq 2000^29^.

### Bioinformatics analysis of miRNAs

The raw reads of small RNAs were first trimmed for adaptor sequences, and the reads with unknown nucleotides (>10%) were removed. For small RNA analysis, only 18–32 nt sequences were used for further study. Then, clean reads of nucleotides were aligned using Rfam v11.0 (http://Rfam.sanger.ac.uk/) to identify miRNA sequences. Besides the annotated miRNAs, novel miRNAs were predicted by miRDeep2 and Randfold^43^. The miRNAs were then aligned to zebrafish genome sequence (zv9, ftp://ftp.ensembl.org/pub/release-77/fasta/danio_rerio/dna/). The relative miRNA expression levels were estimated using the DESeq method^44^. To determine difference significance among the tested samples, the adjusted *P-values* obtained by Benjamini-Hochberg correction were deployed for controlling the FDR. At *P-values* <0.05, the differential expression was confirmed. Prediction of miRNA target genes was performed by Miranda (http://www.miRNA.org/miRNA/home.do)^45^.

## Additional Information

**Accession codes:** Short read sequences were deposited in the NCBI Sequence Read Archive with a study number SRX798682, SRX798683, SRX1034087, SRX1034088, SRX1034089, and SRX1034090.

**How to cite this article**: Zhou, Y. *et al.* Global transcriptional and miRNA insights into bases of heterosis in hybridization of Cyprinidae. *Sci. Rep.* **5**, 13847; doi: 10.1038/srep13847 (2015).

## Supplementary Material

Supplementary Information

Supplementary Dataset 1

Supplementary Dataset 2

Supplementary Dataset 3

Supplementary Dataset 4
