# Supplementary material for: Global transcriptional and miRNA insights into bases of heterosis in hybridization of Cyprinidae
Source: Sci Rep. 2015 Sep 8;5:13847. doi: 10.1038/srep13847 (PMC4561955; doi:10.1038/srep13847)
Supplement: Supplementary Information [file srep13847-s1.pdf]

Supplementary Information

Global transcriptional and miRNA insights into bases of heterosis in hybrid speciation of Cyprinidae

Yi Zhou[1,2¶], Li Ren[1¶], Jun Xiao[1¶], Huan Zhong[1,2¶], Jun Wang[1], Jie Hu[1], Fan Yu[1], Min Tao[1], Chun Zhang[1], Yun Liu[1], Shaojun Liu[1,*]

1. Key Laboratory of Protein Chemistry and Developmental Biology of the State Education Ministry of China, College of Life Sciences, Hunan Normal University, Changsha 410081, China

2. Guangxi Key Laboratory of Aquatic Genetic Breeding and Healthy Aquaculture, Guangxi Academy of Fishery Sciences, Nanning 530021, Guangxi, China

[*] Corresponding author:

Prof. Shaojun Liu, Key Laboratory of Protein Chemistry and Developmental Biology of the State Education Ministry of China, College of Life Sciences, Hunan Normal University, Changsha 410081, China.

Tel: +86 731 88873010

Fax: +86 731 88873074

E-mail: lsj@hunnu.edu.cn

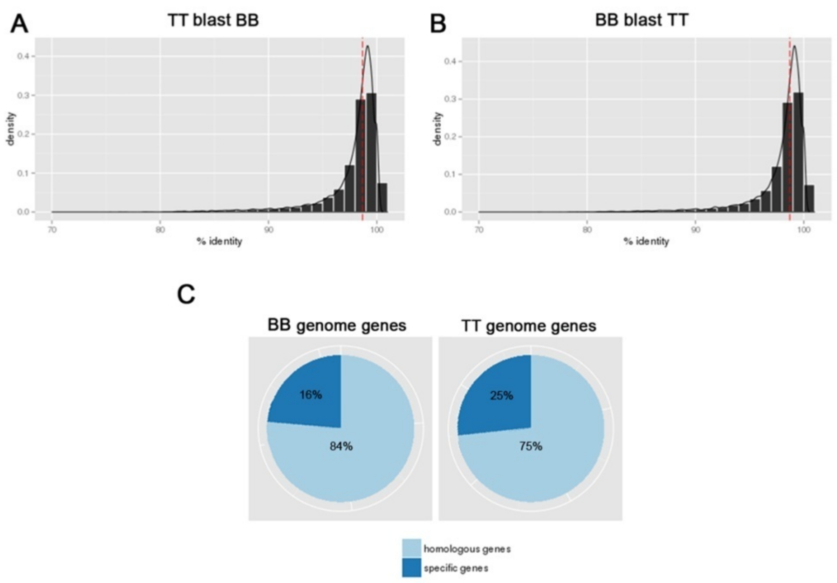

Supplementary Figure 1 Comparative analysis of BB and TT. A and B. Distribution of sequence identity derived from bi-directional BlastN search of BB and TT. C. Percentage distribution of genome specific genes between BB and TT (dark blue).

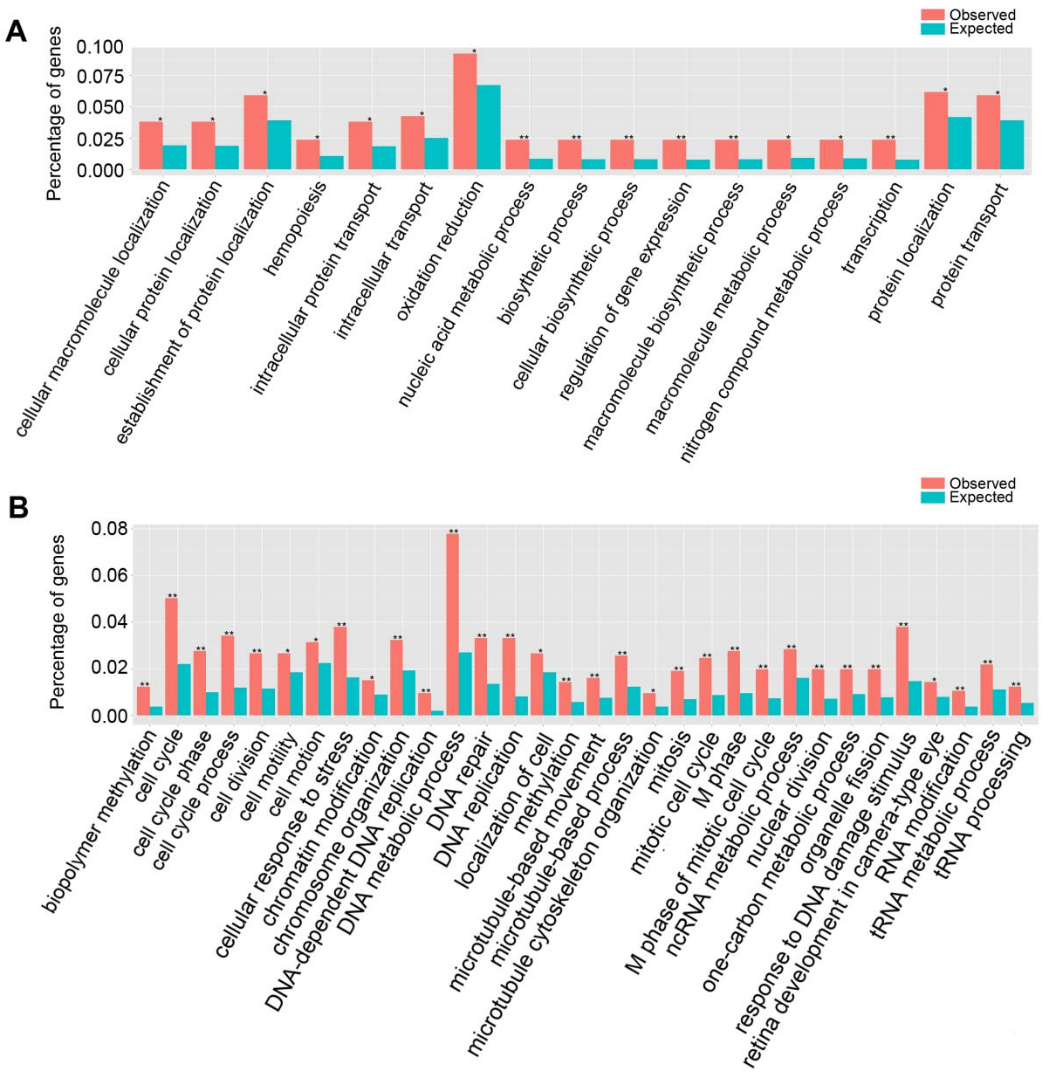

Supplementary Figure 2 Functional categories of genes showing specific expression in BB (A) and TT (B). FDR-adjusted P values, *P < 0.05 and **P < 0.01, respectively. Observed, numbers of genes observed in this study; Expected, numbers of genes in this same category in the GO enrichment analysis program.

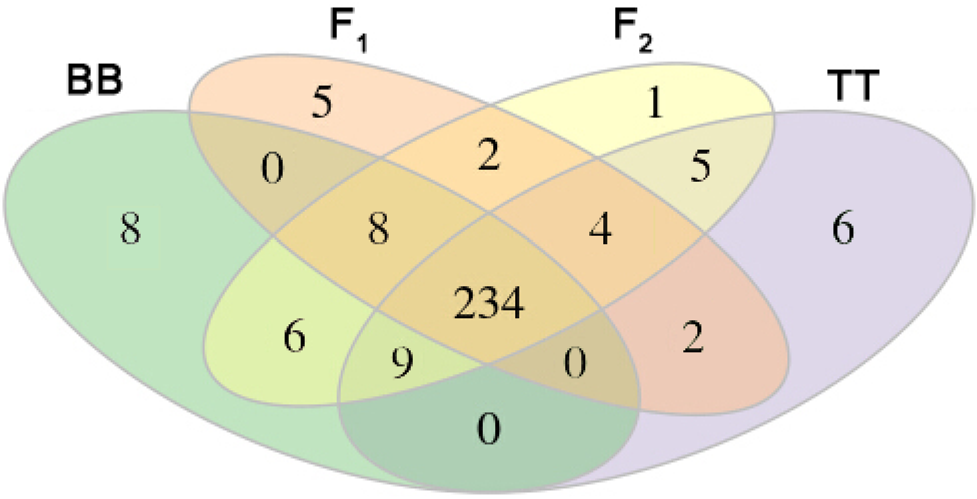

Supplementary Figure 3 Venn diagram analyses of tissue specific miRNAs in hybrids and their parents.

Supplementary table 1 RNA-seq Data Statistics and annotation

	Clean Reads	Contigs	Unigenes	Matching to databases						
				NR	NT	Swiss-Prot	KEGG	COG	GO	ALL
M. amblycephala	55,286,102	105,730	41,825	24,856	35,150	22,172	17,936	6,987	17,806	35,878
C. alburnus	54,896,738	96,499	47,663	29,206	40,416	25,918	21,031	8,481	21,127	41,324
F_1	55,330,970	81,796	43,758	28,537	37,691	25,286	20,401	8,345	20,454	38,482
F_2	55,289,908	82,167	47,835	31,791	41,277	28,378	22,653	9,470	22,525	42,205

Supplementary table 2 Differentially expressed genes among hybrids F_1, F_2 and progenitors.

	Total	BB=TT	BB>TT	BB<TT
**Comparison between the parents**				
BB VS TT	5566	1854	1758	1954
**Comparison between hybrids and the parents**				
F_1 > TT	3336	1208	1268	860
F_1 < TT	1046	179	216	651
F_1 > BB	3417	1198	654	1565
F_1 < BB	1024	171	705	148
F_2 > TT	4207	1517	1417	1273
F_2 < TT	813	114	231	468
F_2 > BB	4241	1528	1063	1650
F_2 < BB	789	117	492	180
**Regulation in hybrids**				
additively expressed genes in F_1 (= MPV)	1313	482	402	429
nonadditively expressed genes in F_1 (≠ MPV)	4253	1372	1356	1525
upregulated in F_1 (>MPV)	3262	1199	945	1118
downregulated in F_1 (<MPV)	991	173	411	407
additively expressed genes in F_2 (= MPV)	555	200	181	174
nonadditively expressed genes in F_2 (≠ MPV)	5011	1654	1577	1780
upregulated in F_2 (>MPV)	4195	1537	1236	1422
downregulated in F_2 (<MPV)	816	117	341	358

Note: MPV were calculated as (BB+TT)/2

Supplementary table 3 The 12 differential expression categories of miRNAs among hybrids F_1, F_2 and progenitors.

Organism	Additively expressed		ELD-B		ELD-T		Transgressive downregulation			Transgressive upregulation		
	I	XII	II	XI	IV	IX	III	VII	X	V	VI	VIII
F_1	38	29	6	10	10	23	40	7	31	7	19	6
F_2	30	23	3	11	8	12	48	11	36	12	22	5

Roman numerals indicate the same categorization as used in Fig.4, with figures schematizing their respective miRNA expression pattern for BB, TT, F_1, and F_2.
